# Regulatory Role of Long Noncoding RNAs LINC00449 in Hepatocellular Carcinoma Mechanism and Prognosis Via Sponge miR-329-3p/KIF5A Axis

**DOI:** 10.5152/tjg.2024.24297

**Published:** 2024-11-04

**Authors:** Yangyang Tang, Jinhuang Lin, Xiaobin Chi, Yichuan Zhang

**Affiliations:** 1 Department of General Surgery, Shanghai General Hospital, Shanghai Jiao Tong University School of Medicine, Shanghai, China; 2 Department of Surgery, Zhangzhou Municipal Hospital of Fujian Province, Zhangzhou, China; 3 Department of Hepatobiliary Surgery, 900 Hospital of the Joint Logistics Support Force, Fuzhou, China; 4 Minimally Invasive Endoscopy Center, Digestive Disease Center, The Affiliated Hospital of Panzhihua University, Panzhihua, China

**Keywords:** Hepatocellular carcinoma,, prognosis,, LINC00449,, miR-329-3p,, KIF5A

## Abstract

**Background/Aims:**

Hepatocellular carcinoma (HCC) is a globally prevalent malignant tumor with high recurrence and mortality rates. Long noncoding RNAs (lncRNAs) have been utilized to investigate the progression of various cancers, including HCC. The objective of this research is to explore the mechanism and prognostic impact of lncRNA LINC00449 on HCC.

**Materials and Methods:**

LINC00449 and miR-329-3p levels in HCC were measured using RT-qPCR. The impact of high- or low-LINC00449 expression on survival was tested. CCK-8, plate cloning, flow cytometry, and Transwell assays were selected to assess the biological functions of HCC cells. Kaplan–Meier and multivariate Cox analyses were conducted to evaluate the potential of LINC00449 as a prognostic marker for HCC. The interaction between LINC00449, miR-329-3p, and KIF5A was investigated through luciferase activity assays.

**Results:**

LINC00449 expression in HCC tissues was increased. Silencing of LINC00449 improved the survival prospects of patients with HCC. Moreover, LINC00449 targeting the miR-329-3p/KIF5A axis influences the progression of HCC.

**Conclusion:**

LINC00449 may become a biomarker for the prognosis of HCC, and the LINC00449/miR-329-3p/KIF5A regulatory network mediates the deterioration of HCC.

**Main Points**

- The expression of LINC00449 was upregulated in HCC.
- Low expression of LINC00449 contributed to the survival of patients.
- LINC00449 has potential as a prognostic marker for HCC.
- LINC00449 inhibited the biological functions of HCC cells by targeting the miR-329-3p/KIF5A axis.

## Introduction

Liver cancer incidents and fatalities are alarmingly high, with an increasing number of patients being diagnosed each year in China. Our research primarily focuses on hepatocellular carcinoma (HCC), a type of primary liver cancer.^1,2^ Patients with HCC have a poor prognosis due to high recurrence and metastasis rates.^3^ Currently, the origins and the underlying mechanisms of HCC have yet to be fully established. It is recognized that the occurrence of HCC is influenced by factors such as genetics, chemical carcinogens, and smoking. The modern medical treatment for HCC mainly adopts surgical methods, like resection and transplantation, but the probability of metastasis and recurrence in patients reaches 60 -70% within 5 years.^4-6^

Relevant researchers have used long noncoding RNAs (lncRNAs) to study the pathogenesis and progression of multiple cancers, including HCC. Nucleotides with transcripts larger than 200 bases that do not translate into protein are defined as lncRNAs.^7^ These can perform various functions such as transcriptional regulation and RNA molecular regulation. Existing evidence has revealed the different effects of lncRNAs in digestive tumors, reproductive system diseases, and liver diseases.^8-10^ For reports of lncRNAs in HCC, Wu et al^11^ stated that 4 lncRNAs related to autophagy can predict HCC prognosis through co-expression and ceRNA mechanisms. Wang et al^12^ confirmed that lncRNA MCM3AP-AS1 is a new oncogenic lncRNA, which can contribute to HCC by targeting miR-194-5p. Importantly, it has been pointed out that LINC00449, a novel lncRNA, may have prognostic potential and regulatory capacity in various tumors.^13^ Meanwhile, LINC00449 was considered a prospective therapeutic target in triple-negative breast cancer^14^ and gastric cancer.^15^ However, the biological and clinical role of lncRNA LINC00449 in HCC requires further in-depth study.

This paper aims to determine the prognostic significance of the LINC00449/miR-329-3p/KIF5A regulatory network in HCC, which was identified for the first time by investigating the changes in the biological activity of HCC cells after the knockdown of LINC00449 and its mechanism of targeting downstream factors. In this way, we may find effective prognostic biomarkers of HCC and assist patients in realizing the monitoring of their condition.

## Materials and Methods

### Patient Clinical Data and Tissue Samples

This study randomly selected 128 HCC patients from the 900 Hospital of The Joint Logistics Team as experimental subjects. Under the supervision of professional histopathologists, HCC tissues from patients were stored in liquid nitrogen at −80°C to maintain the tissues’ health. The relevant clinical data of HCC patients and the expression of LINC00449 are detailed in Table 1. The inclusion requirements of 128 HCC patients were as follows: patients clinically diagnosed with HCC by oncologists; patients who did not receive any preoperative treatment before surgery; and none of the patients had any family genetic disease or other serious diseases. Exclusion criteria: patients with other serious diseases that may affect the study results; patients who are unable to understand the purpose of the study or follow the study procedures; and patients who were also enrolled in another clinical trial or had incomplete clinical data.

This study was approved by the research Ethics Committee of the 900 Hospital of The Joint Logistics Team (approval no: 2016-014, date: October 18, 2016), and all patients provided written consent. Through telephone follow-ups, face-to-face patient interviews, and communication with family members, the clinical data over a 5-year period were obtained.

### Cultivation and Growth of Cells

Cell lines (SK-Hep-1, Huh-7, SMCC-7721, MHCC97-H, and the control LO2) were maintained in Dulbecco’s modified Eagle medium (DMEM) (Invitrogen, USA) with 10% fetal bovine serum (FBS). The cells were placed in an incubator with 5% CO_2_ at 37°C.

Lipofectamine 2000 (Invitrogen, USA) is available for cell transfection. Both SMCC-7721 and SK-Hep-1 cells were transfected with silencing LINC00449 (si-LINC00449), negative control (si-NC), si-LINC00449+miR NC, si-LINC00449+miR-329-3p inhibitor, si-LINC00449+miR-329-3p inhibitor+mRNA NC, si-LINC00449+miR-329-3p inhibitor+si-KIF5A, or control.

### Real-Time Quantitative Polymerase Chain Reaction Assay

RNA was extracted with TRIZOL reagent. Purity and concentration tests were immediately conducted to ensure RNA quality. The RNA was converted to cDNA using the reverse transcription kit (Invitrogen, USA). Next, the SYBR-Green kit (Takara, Japan) was used in the Bio-Rad CFX96 system to perform the RT-qPCR assay. Lastly, the data were normalized to GAPDH or U6 and calculated using the 2^−ΔΔCt^ method.

### Cell Counting Kit-8 Assay

SMCC-7721 and SK-Hep-1 cells were cultured at a cell density of 1 × 10^3^/mL in 96-well plates. At 0, 24, 48, 72, and 96 hours, 10 μL of CCK-8 solution (Dojindo, Japan) were added to each well. After 4 hours of incubation under the same culture conditions, the absorbance was measured using a microplate spectrophotometer (Thermo, USA).

### Plate Cloning Assay

SMCC-7721 and SK-Hep-1 cells were inoculated into a 6-well plate filled with DMEM medium. After 14 days of growth, the cells were stained with crystal violet and examined under a microscope.

### Cell Apoptosis Assay

After washing SMCC-7721 and SK-Hep-1 cells with PBS, 5 μL of Annexin V-FITC and PI reagent (BD Biosciences, USA) were added and mixed evenly. The assessment of apoptosis levels was conducted via flow cytometry analysis following 15 minutes of continuous culture.

### Transwell Assay

The procedures for cell migration and invasion were essentially the same, except for the addition of Matrigel (BD Biosciences, USA) in the Transwell upper chamber (Millipore, USA) for the invasion assay. The specific steps include culturing cells in the upper part of the Transwell chamber, placing DMEM medium and 10% FBS in the lower part, fixing the cells in 4% paraformaldehyde after 48 hours of incubation, and staining with crystal violet. Finally, the cells were observed under the microscope.

### Luciferase Activity Assay

Downstream targets of LINC00449 and miR-329-3p were screened through the LncBook 2.0 online database, and then a luciferase activity assay was conducted for further verification. The LINC00449 sequence was cloned into the pmirGLO vector (Promega, USA), with miR-329-3p wild-type (WT) and mutant-type (MUT) binding sites to construct WT-LINC00449 and MUT-LINC00449. Subsequently, luciferase activity was examined in SMCC-7721 cells co-transfected with either WT-LINC00449 or MUT-LINC00449, and miR-329-3p mimic or mimic NC. The dual-luciferase reporter gene analysis system (Promega, USA) was utilized to detect the luciferase activity. Similarly, the connection between miR-329-3p and KIF5A was confirmed after the construction of WT-KIF5A and MUT-KIF5A.

### Statistical Analysis

The data were analyzed using SPSS software version 26 (IBM SPSS Corp.; Armonk, NY, USA). The differences in count data between groups were analyzed by the chi-square test. The Student’s *t*-test or one-way ANOVA was used to calculate the difference between 2 or more groups of measurement data. The relationship between LINC00449 and miR-329-3p levels was determined by Pearson correlation analysis. The impact of LINC00449 expression on the clinical characteristics of HCC patients was assessed by multivariate Cox regression analysis, and the survival probability was determined by the Kaplan–Meier method. Each group was set up with 3 parallel experiments and repeated at least 3 times. There is significant meaning when the *P-*value is less than .05.

## Results

### LINC00449 and miR-329-3p Expression in Hepatocellular Carcinoma Tissue

**Figure 1.**
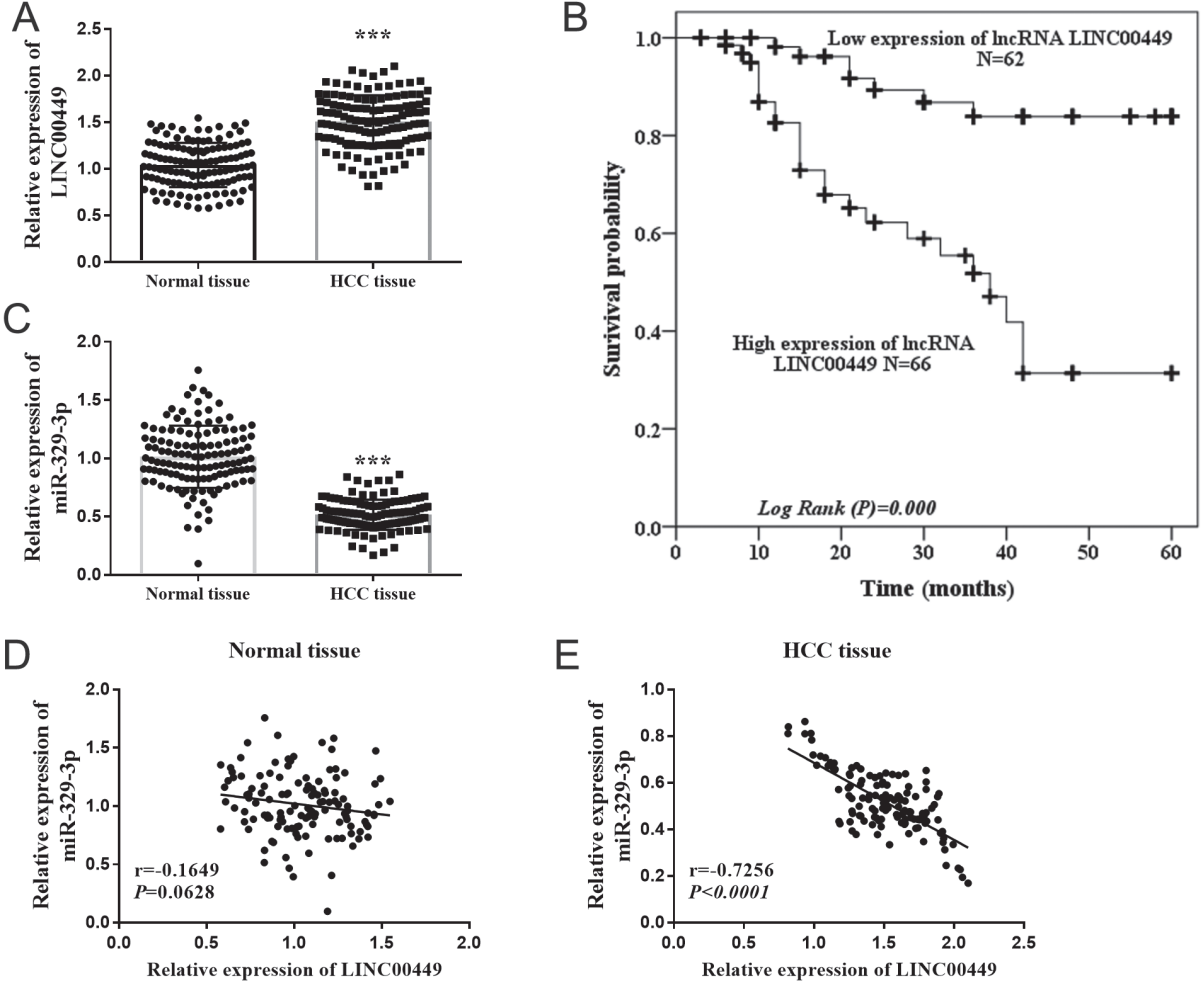
The expression and interaction of LINC00449 and miR-329-3p in HCC tissues. (A) LINC00449 level is upregulated in HCC tissues (^***^P < .001). (B) The survival probability of HCC patients with low-LINC00449 expression is higher than high-LINC00449 expression (*P *= .000). (C) miR-329-3p level is downregulated in HCC tissues (^***^P < .001). (D) In normal tissues, there is no significant correlation between LINC00449 and miR-329-3p (*r* = −0.1649, *P *= .0628). (E) In HCC tissues, LINC00449 and miR-329-3p have a negative interaction (*r* = −0.7256, *P *< .0001).

Figure 1A describes that LINC00449 levels in HCC tissue were higher than in normal tissues. Hepatocellular carcinoma patients were categorized into high- (n = 66) and low-level (n = 62) groups based on the average LINC00449 level, with their survival status displayed in Figure 1B. Kaplan–Meier curve indicated that lower LINC00449 expression may be more conducive to the survival of HCC patients within 5 years (log-rank *P *= .000). Figure 1C illustrates a decrease of miR-329-3p in HCC tissues. Furthermore, LINC00449 and miR-329-3p revealed no significant correlation in normal tissues (*r* = −0.1649, *P *= .0628, Figure 1D), but a negative interaction in HCC tissues (*r* = −0.7256, *P *< .0001, Figure 1E) through Pearson correlation analysis.

### The Influence of LINC00449 Expression on the Clinical and Prognosis of Hepatocellular Carcinoma Patients

The clinical characteristics of all 128 HCC patients are recorded in Table 1. Chi-square analysis expounds that LINC00449 expression was affected by tumor size (*P *= .048), vascular invasion (*P *= .032), and TNM stage (*P *< .001).

The prognostic factors of HCC include LINC00449 (*P *= .007), vascular invasion (*P *= .038), and TNM stage (*P *= .033) using multivariate Cox analysis. Thus, LINC00449 potentially influences the prognosis of HCC (Table 2).

### Low Expression of LINC00449 Inhibited the Growth of Hepatocellular Carcinoma Cells

**Figure 2.**
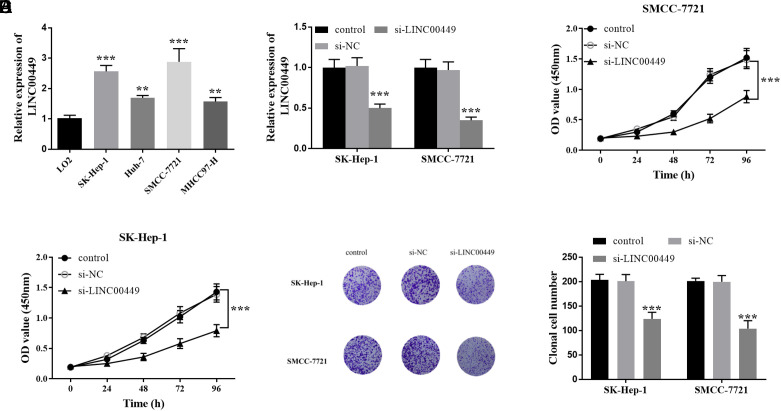
The effect of silencing LINC00449 on hepatocellular carcinoma cell proliferation. (A) LINC00449 is enhanced in SK-Hep-1, Huh-7, SMCC-7721, and MHCC97-H cells (^**^P < .01, ^***^P < .001). (B) Transfection efficiency of si-LINC00449 in SMCC-7721 and SK-Hep-1 cells (^***^P < .001). (C) and (D) The proliferation level of cells after transfection with si-LINC00449 (^***^P < .001). (E) The proliferation level of cells was verified by plate cloning assay (^***^P < .001) (magnification, ×200).

**Figure 3.**
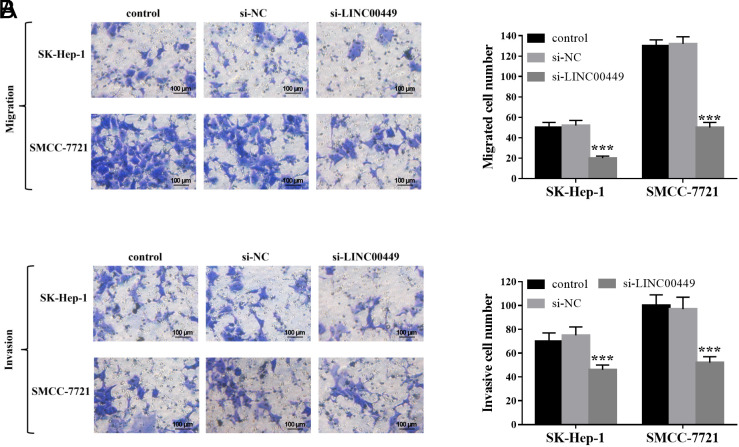
The assay of transwell migration and invasion. (A) The migration ability of cells after transfection with si-LINC00449 (^***^P < .001) (magnification, ×200). (B) The invasion ability of cells after transfection with si-LINC00449 (^***^P < .001) (magnification, ×200).

LINC00449 levels were upregulated in the HCC cells. Among them, LINC00449 increased more significantly in SMCC-7721 and SK-Hep-1 cells (Figure 2A). Figure 2B indicates that LINC00449 was markedly downregulated in these cells by si-LINC00449. Additionally, the OD value of si-LINC00449 at 450 nm decreased, implying a reduction in the HCC cells’ proliferation ability (Figure 2C and D). The plate cloning assay demonstrated that si-LINC00449 reduced the number of cell clones in Figure 2E. Figure 3A presents the number of migrating cells detected, indicating a weakened level of cell migration. Similarly, transfection of si-LINC00449 resulted in reduced invasiveness of HCC cells (Figure 3B).

### LINC00449 Targeted miR-329-3p

**Figure 4.**
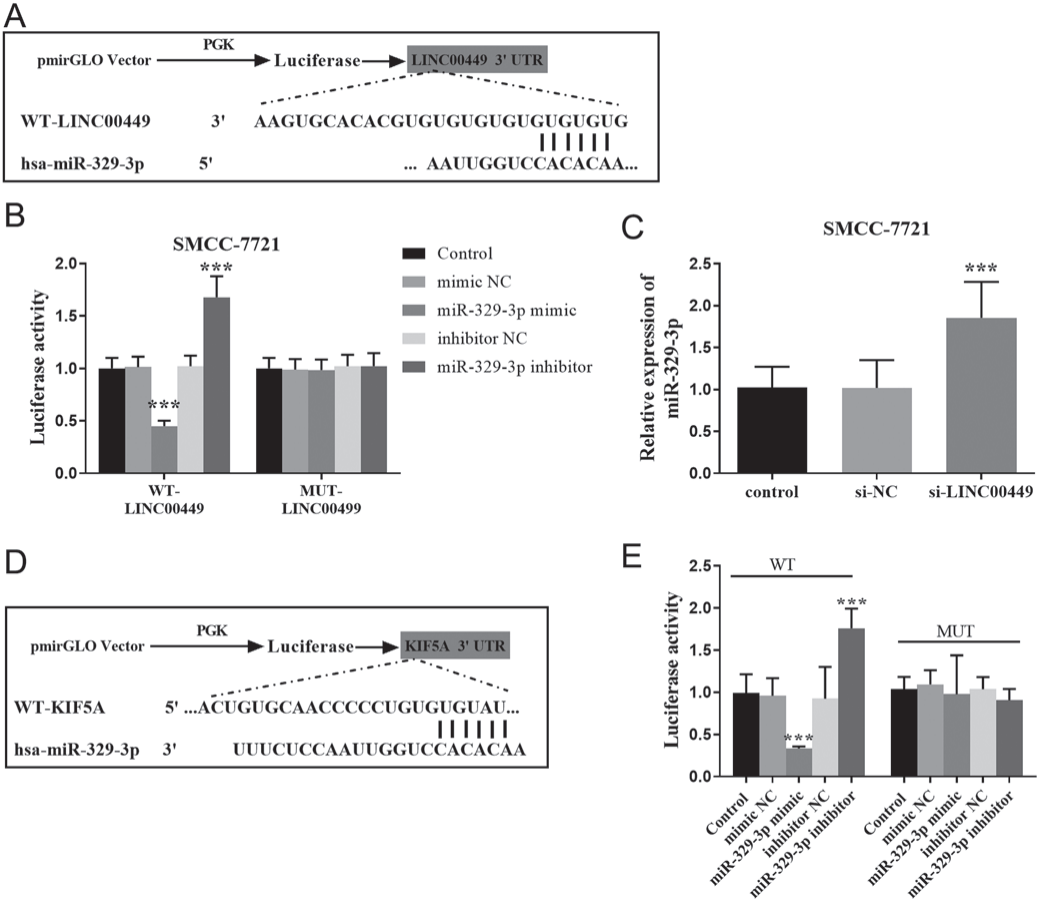
Bioinformatics analysis and luciferase activity assays. (A) Binding sites of WT-LINC00449 and miR-329-3p. (B) Luciferase activity of SMCC-7721 cell (^***^P < .001). (C) Silencing of LINC00449 increased miR-329-3p expression in SMCC-7721 cells (^***^P < .001). (D) There are link sites between miR-329-3p and KIF5A 3’-UTR. (E) Changes in luciferase activity after co-transfection of WT-KIF5A and MUT-KIF5A (^***^P < .001).

The luciferase activity and the LINC00449 levels were determined in SMCC-7721 cells. According to Figure 4A, miR-329-3p and LINC00449 have binding sites. Figure 4B shows that the miR-329-3p mimic downregulated the luciferase activity of WT-LINC00449, while the miR-329-3p inhibitor upregulated it. Transfection with si-LINC00449 upregulated miR-329-3p expression, reinforcing that LINC00449 negatively correlates with miR-329-3p (Figure 4C).

### KIF5A Was the Target Site of miR-329-3p

To delve deeper into the regulatory network of LINC00449 in HCC, Figure 4D exhibits the presence of complementary bases between miR-329-3p and KIF5A 3’-UTR. Additionally, the luciferase reporter assay confirmed KIF5A as a downstream target of miR-329-3p in Figure 4E. After transfecting the miR-329-3p mimic, the luciferase activity decreased in the WT group.

### LINC00449/miR-329-3p/KIF5A Regulatory Network in Hepatocellular Carcinoma

**Figure 5.**
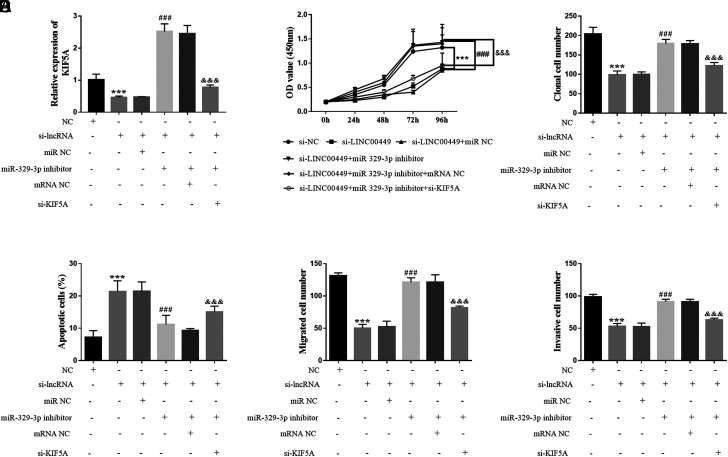
Roles of miR-329-3p and KIF5A in hepatocellular carcinoma. (A) Different results of transfection in cells. (B) and (C) The effect of co-transfection of miR-329-3p inhibitor and si-KIF5A on cell proliferation. (D) The apoptosis rate of HCC cells. (E) and (F) The number of cell migrations and invasions after co-transfection of miR-329-3p inhibitor and si-KIF5A. (^***^P < .001, compared with NC group; ^###^*P *< .001, compared with si-LINC00449 group; ^&&&^*P* < .001, compared with si-LINC00449+miR-329-3p inhibitor group).

Transfection assays revealed that KIF5A content was downgraded after transfection with si-LINC00449. Transfection with si-LINC00449+miR-329-3p inhibitor significantly upregulated KIF5A levels. Transfection with si-LINC00449, miR-329-3p inhibitor, and si-KIF5A restored KIF5A expression (Figure 5A). The number of cell proliferations increased after co-transfection of si-LINC00449+miR-329-3p inhibitor, while the participation of si-KIF5A played an inhibitory function, as displayed in Figure 5B and C. In addition, the apoptosis assays in Figure 5D elucidated that si-LINC00449 induced apoptosis in the cells. Transfection with miR-329-3p inhibitor reduced the apoptosis rate, whereas the addition of si-KIF5A restored the apoptosis rate. Furthermore, the high metastatic ability (Figure 5E and F) of the cells was reversed by si-KIF5A transfection. In conclusion, both miR-329-3p and KIF5A were confirmed to partake in the LINC00449 regulatory network in HCC.

## Discussion

In recent decades, there has been a noted increase in the prevalence and mortality of HCC.^16^ The available treatment methods and drugs for HCC are limited and subject to genetic constraints.^17,18^ Current studies show that a combination of immune checkpoint inhibitors with kinase inhibitors, anti-angiogenic drugs, or chemotherapy has a positive impact on HCC patients’ prognosis.^19^ Therefore, it is crucial to conduct in-depth research on the mechanisms related to its pathogenesis and discover more effective treatments for HCC.

Existing research has proved that lncRNA participates in the process of HCC by targeting miRNA, potentially serving as a target for tumor therapy. For instance, lncRNA ZFPM2‑AS1 targeted the miR-3065-5p/XRCC4 axis to mediate HCC progression.^20^ The LncRNA SNHG20/miR-5095/MBD1 regulatory network influenced HCC cell metastasis and apoptosis, providing fresh insights into HCC growth.^21^ Additionally, Ping et al^14^ confirmed that reducing the expression of LINC01270 and LINC00449 can diminish the viability, migration level, and colony-forming ability of triple-negative breast cancer cells, indicating that LINC00449 and LINC01270 have the potential to become prognostic biomarkers of TNBC. Zhang and colleagues^15^ also believed that LINC00449 has potential for treatment and prognosis in gastric cancer patients. Consequently, lncRNA LINC00449 may also have the possibility of regulating the development and prognosis of HCC by binding to downstream factors. This study revealed that LINC00449 was upregulated in HCC, and this trend inhibited patient survival. In existing reports, lncRNA FTO-IT1, METTL3, and HOXD-AS2 were all stated to be upregulated in HCC and associated with a poor prognosis of patients, which is similar to our results.^22-24^ To better understand the characteristics and mechanism of low LINC00449 expression, the impact of dysregulated LINC00449 on the biological function of HCC cells was clarified. This study revealed that the expression of LINC00449 was elevated in HCC cells through in vitro cell assays, and silencing LINC00449 obviously inhibited the biological function of HCC cells. This suggests that low levels of LINC00449 are detrimental to cell activity and tumor development. Meanwhile, the targeting effect of LINC00449 on miR-329-3p was confirmed by bioinformatics verification and a luciferase activity assay. Additionally, miR-329-3p was indicated to target KDM1A and inhibit the immunosuppression of HCC cells.^25^ USP22 was also a targeting factor of miR-329-3p, which attenuated the activity of HCC cells by regulating the USP22-Wnt/β-Catenin pathway.^26^ Furthermore, the recovery assay revealed that the miR-329-3p inhibitor restored the suppressive effects of silencing LINC00449 on cell proliferation, apoptosis, migration, and invasion. Through studying HCC tissues and cells, there is good reason to believe that LINC00449 targeting the miR-329-3p affects the prognosis of HCC patients, offering potential research value as a cancer-promoting factor.

KIF5A is a microtubule-based motor protein involved in intracellular protein transport. As a gene closely related to amyotrophic lateral sclerosis, KIF5A has received extensive attention.^27^ Concurrently, it has been proposed that KIF5A is related to poor prognosis in various tumors. Guo et al^28^ have highlighted that the miR-329-3p/FOXK1 axis of TPCO-AS1 sponge influences the occurrence and development of HCC. This led us to surmise that the LINC00449/miR-329-3p axis may mediate downstream factors to regulate tumor growth. Luciferase reporter assay and co-transfection recovery assay confirmed that KIF5A is the target of miR-329-3p, and silencing KIF5A counteracted the promotive effect of the miR-329-3p inhibitor on cell growth.

Undeniably, there are some shortcomings in this study. Enhancing the sample size and diversity of the sample can further improve the persuasiveness of this study. Improving the analysis methods of experimental data and the quality control measures of the experimental scheme can effectively improve the reliability of the results. Supplementing the validation of clinical experiments in vivo and prolonging the follow-up time of clinical samples can provide a more comprehensive understanding of the underlying mechanism of HCC. Furthermore, discussing genomically directed stratifications will be our future focus.

In summary, LINC00449 dysregulation suppressed the growth and proliferation of HCC cells and contributed to the survival of patients. LINC00449 has the potential to serve as a biomarker for HCC and may mediate tumor progression through the LINC00449/miR-329-3p/KIF5A regulatory network.

## Figures and Tables

**Table 1.** Correlation of the Long Noncoding RNA LINC00449 Expression with Clinical Characteristics in Hepatocellular Carcinoma

Indicators	Cases (n = 128)	lncRNA LINC00449 expression		*P*
		Low (n = 62)	High (n = 66)	
Age				.310
≤60	81	42	39	
>60	47	20	27	
Gender				.852
Male	100	48	52	
Female	28	14	14	
Tumor size (cm)				.048
≤2	69	39	30	
>2	59	23	36	
Tumor number				.365
Single	88	45	43	
Multiple	40	17	23	
AFP (ng/mL)				.170
≤400	44	25	19	
>400	84	37	47	
Vascular invasion				.032
Yes	40	25	15	
No	88	37	51	
Cirrhosis				.212
Absence	29	17	12	
Presence	99	45	54	
HBV infection				.229
Absence	15	10	6	
Presence	113	52	60	
TNM stage				
I, II	86	52	34	<.001
III, IV	42	10	32	

**Table 2.** Multivariate Cox Analysis of Clinical Characteristics in Relation to Overall Survival

Indicators	Multivariate Analysis		
	HR	95% CI	*P*
LncRNA LINC00449	3.850	1.450-10.224	.007
Age	1.012	0.445-2.300	.978
Gender	1.229	0.494-3.057	.658
Tumor size	1.670	0.767-3.639	.197
Tumor number	1.567	0.678-3.618	.293
AFP (ng/mL)	2.117	0.728-6.157	.169
Vascular invasion	2.285	1.046-4.990	.038
Cirrhosis	2.107	0.602-7.373	.243
HBV infection	2.011	0.441-9.178	.367
TNM stage	2.747	1.083-6.966	.033

## Data Availability

The data that support the findings of this study are available on request from the corresponding author.
